# Supplementary material for: GABAergic motor neurons bias locomotor decision-making in C. elegans
Source: Nat Commun. 2020 Oct 8;11:5076. doi: 10.1038/s41467-020-18893-9 (PMC7544903; doi:10.1038/s41467-020-18893-9)
Supplement: Supplementary file 1 — Supplementary Information [file 41467_2020_18893_MOESM1_ESM.pdf]

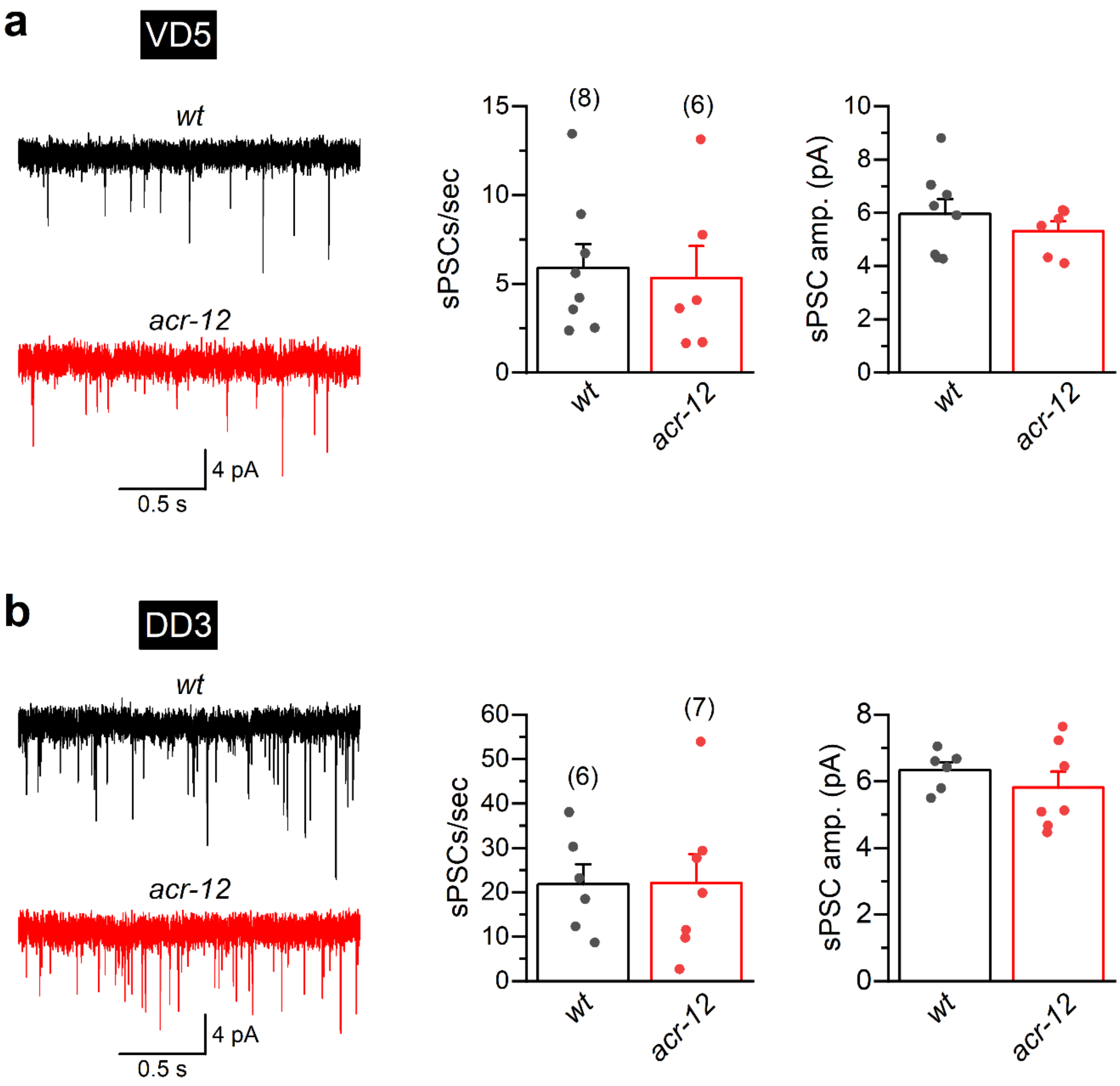

Supplementary Fig. 1. Spontaneous postsynaptic currents (sPSCs) in D-MNs were not compromised in *acr-12* mutant. Voltage-clamp recordings were performed with VD5 (**a**) and DD3 (**b**) of wild type (*wt*) and *acr-12(ok367)*. The holding voltage was -60 mV. The numbers inside brackets indicate sample size (*n*). *n* = numbers of independently recorded cells. Data are presented as mean values ± SEM. Source data are provided as a Source data file.

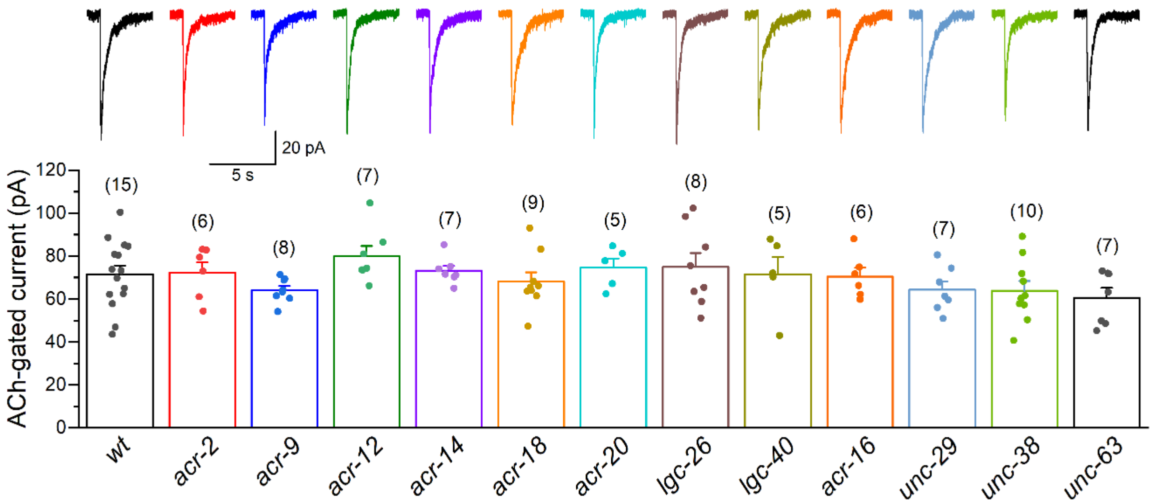

Supplementary Fig. 2. Acetylcholine-induced whole-cell current in VD5 was not compromised in mutants of other candidate acetylcholine receptor genes. Exogenous acetylcholine (100 μM) was pressure-ejected through the tip of a glass pipette aimed at the dendritic area of VD5, which was held at -60 mV. The numbers inside brackets indicate sample size (*n*). *n* = numbers of independently recorded cells. Data are presented as mean values ± SEM. Source data are provided as a Source data file.

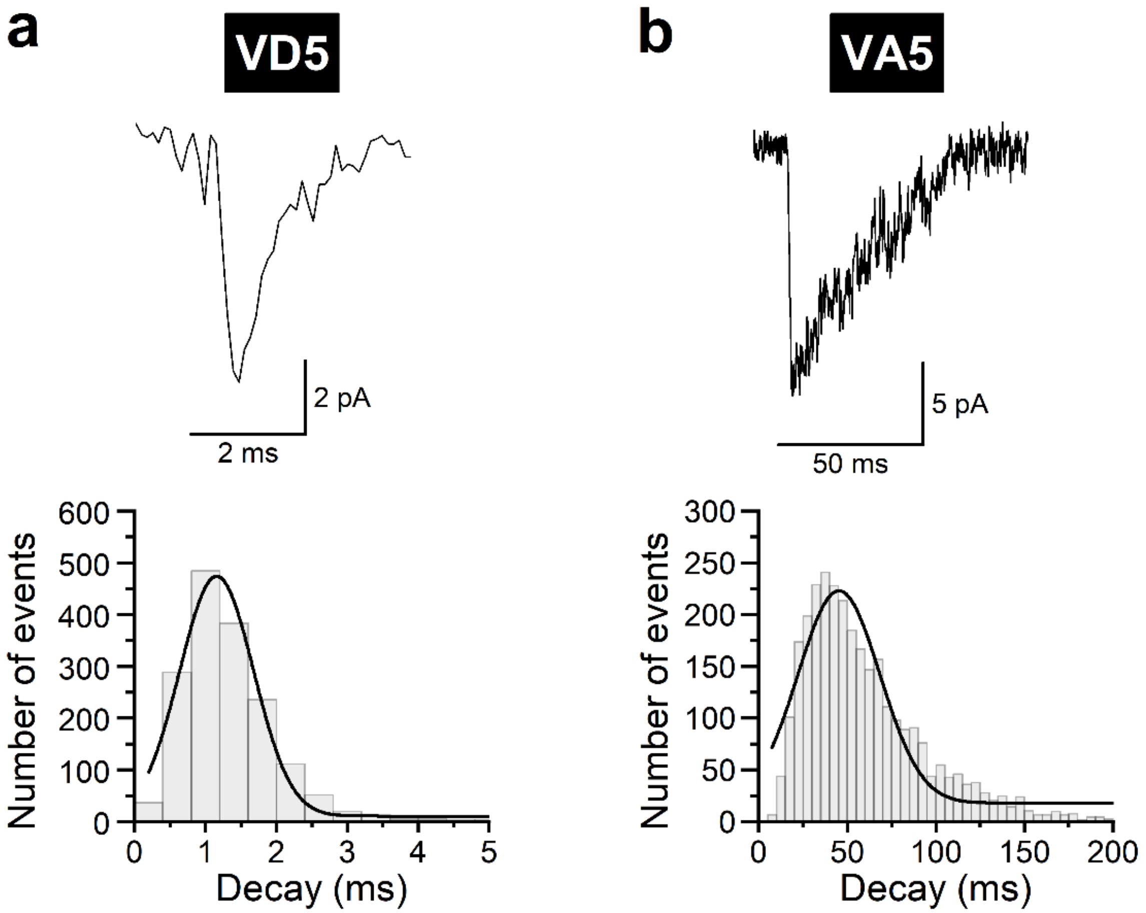

Supplementary Fig. 3. The decay time constant of LGC-46-dependent spontaneous postsynaptic currents (PSC) differed dramatically between VA5 and VD5. The decay time was quantified from fitting the declining phase of spontaneous PSCs to single exponentials. **a,** A representative spontaneous PSC event and a decay time distribution histogram of spontaneous PSCs recorded from VD5 (compiled from the wild-type data in Fig. 1b). **b,** A representative spontaneous PSC event and a decay time distribution histogram of spontaneous PSCs recorded from VA5 (compiled from the large and slow events of wild type in Figure 1 of Liu et al., *Nat Commun* 2017). Source data are provided as a Source data file.

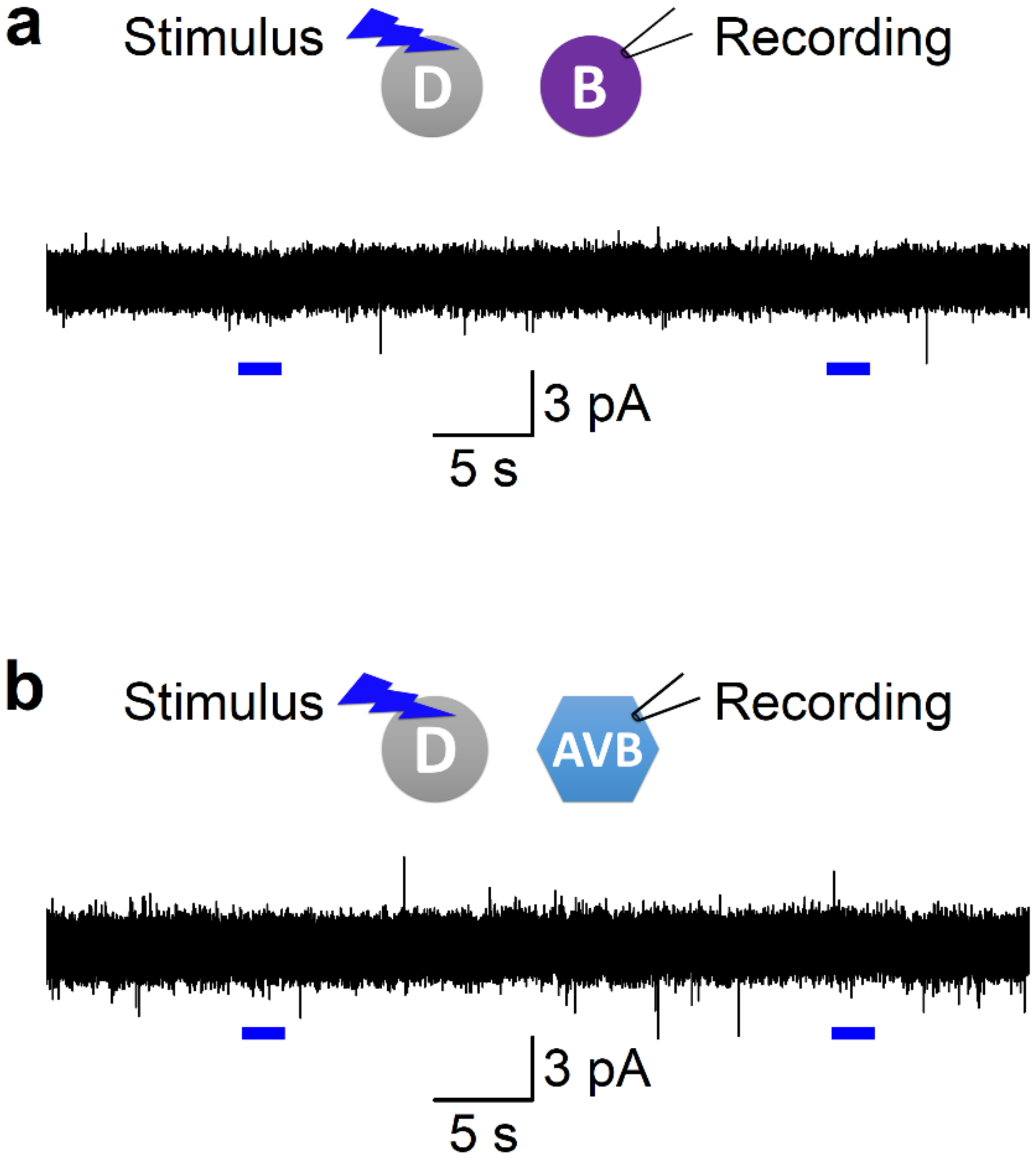

Supplementary Fig. 4. Optogenetic activation of D-MNs did not evoke outward current in either VB6 (a) or AVB (b), which are not innervated by D-MNs. The strain expressing channelrhodopsin-2 under the control of *unc-47* promoter was used in these experiments. The holding voltage was -10 mV. Each sample trace represents similar results from 5 different recordings. Source data are provided as a Source data file.

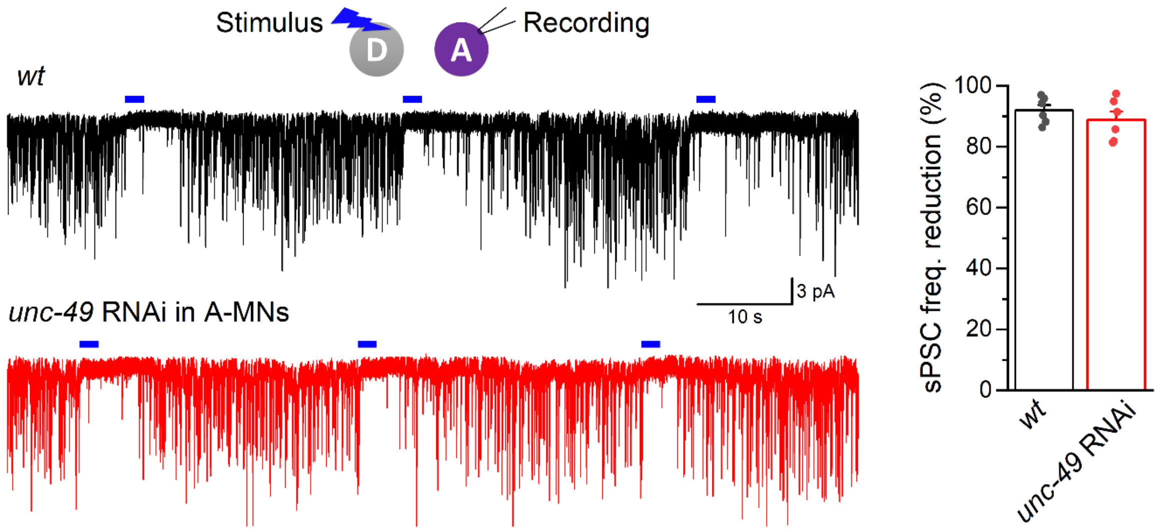

Supplementary Fig. 5. Knockdown of *unc-49* in A-type cholinergic motor neurons (A-MNs) did not compromise the inhibitory effect of optogenetic activation of D-MNs on spontaneous postsynaptic currents (sPSCs) in VA5. The experiments were performed with a strain expressing channelrhodopsin-2 in D-MNs under the control of *unc-47* promoter and *unc-49* RNAi plasmids in A-MNs under the control of *acr-2* promoter. Sample size (*n*) = numbers of independently recorded cells. *n* = 6 in both wild type (*wt*) and *unc-49* RNAi. Data are presented as mean values ± SEM. Source data are provided as a Source data file.

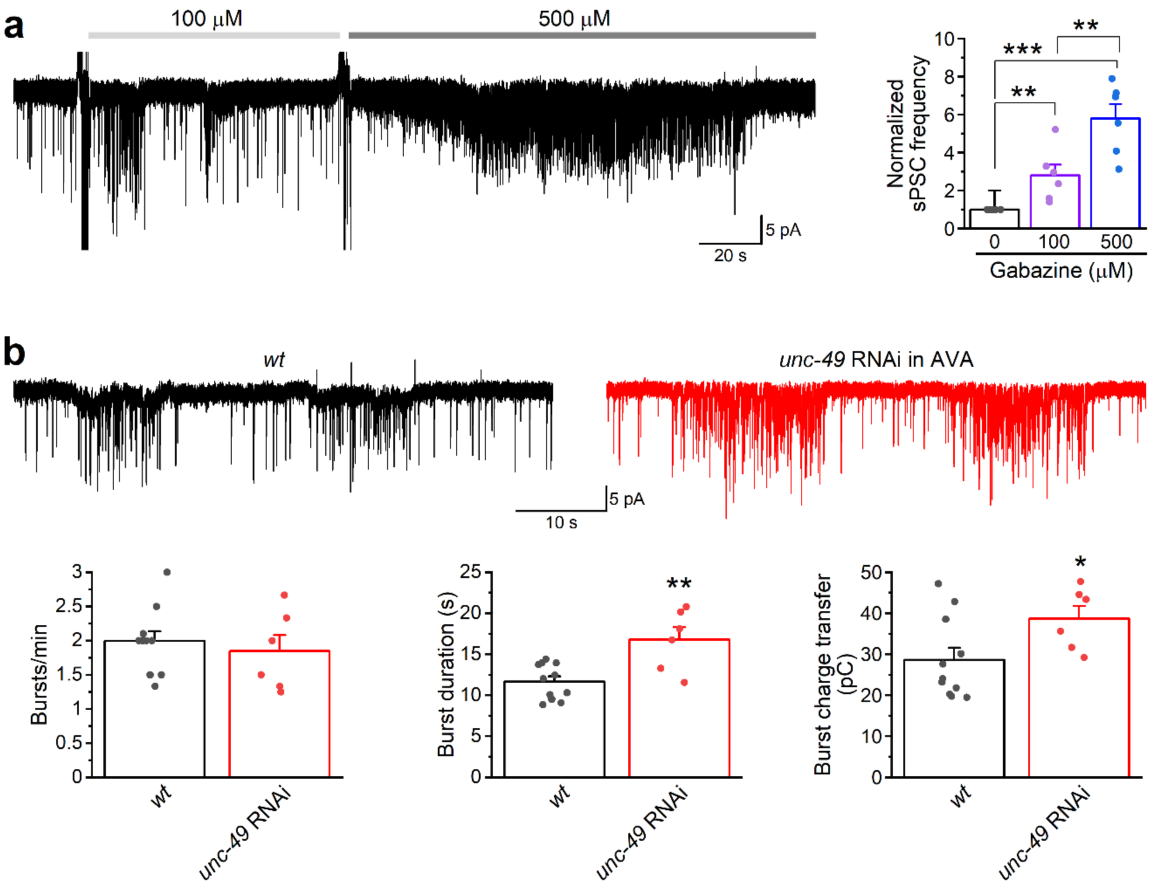

Supplementary Fig. 6. The D-AVA circuit is physiologically important. a, Blockade of the D-AVA circuit by gabazine increased the frequency of spontaneous postsynaptic currents (sPSCs) in VA5 of wild type (*wt*) in a concentration-dependent manner. Gabazine was added to the bath solution through pipetting. *n* = 6. The asterisks indicate significant differences between the indicated gabazine concentrations (** $p < 0.01$, *** $p < 0.001$, one-way repeated measures ANOVA). The *p* values are 0.010 (between 0 and 100 µM), 0.000 (between 0 and 100 µM), and 0.010 (between 100 and 500 µM). **b**, Disruption of the D-AVA circuit by *unc-49* RNAi in AVA increased the duration and total charge transfer of PSC bursts in VA5. Compared with *wt*, *p* = 0.578 Burst frequency, 0.002 Burst duration, and 0.047 Burst charge transfer. Sample size (*n*) = numbers of independently recorded cells. *wt n* = 11. *unc-49* RNAi *n* = 6. The asterisks indicate significant differences compared with *wt* (* $p < 0.05$, ** $p < 0.01$, unpaired two-sided *t*-test). Data are presented as mean values ± SEM. Source data are provided as a Source data file.

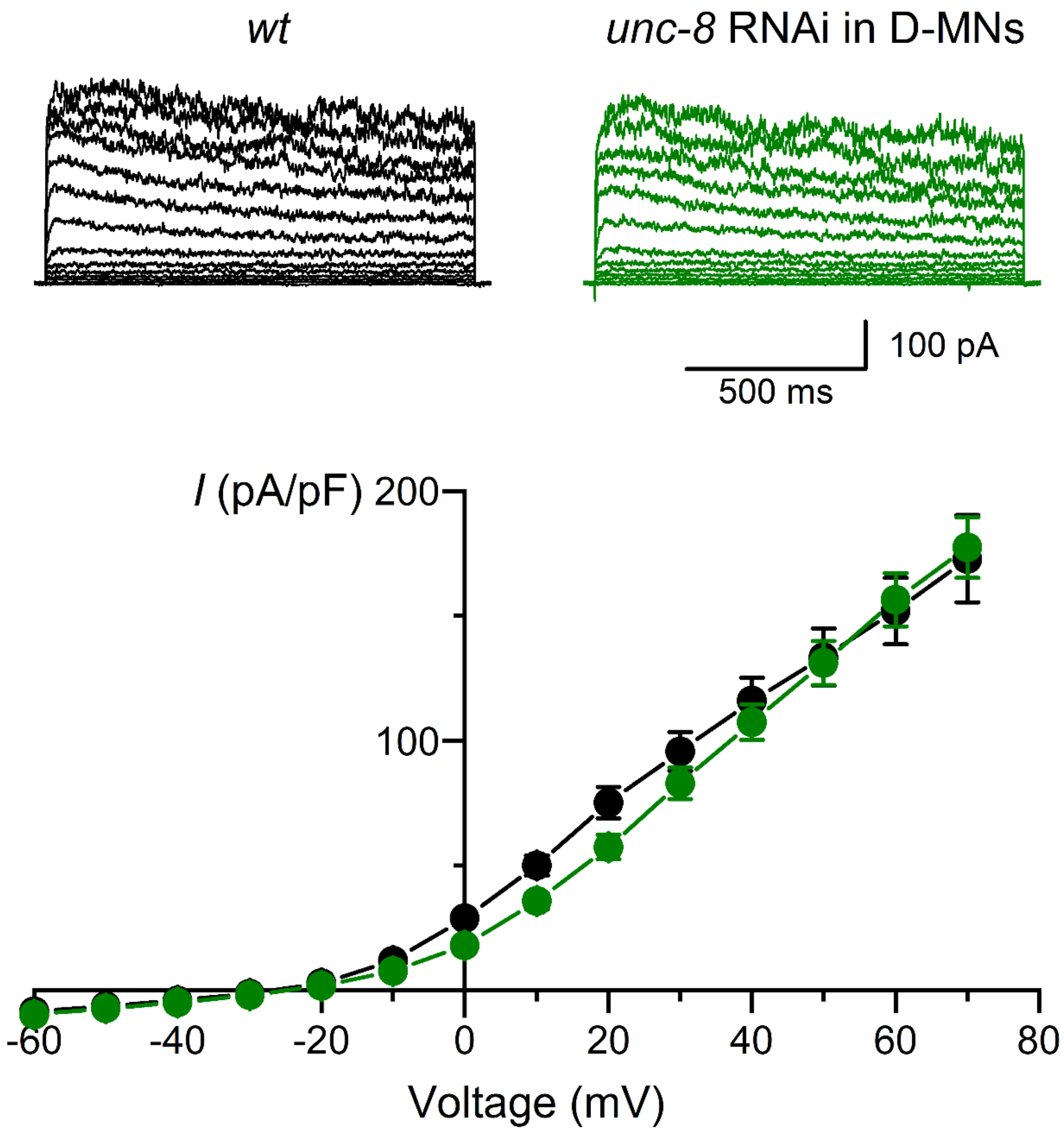

Supplementary Fig. 7. Voltage-dependent current in D-MNs are not affected by UNC-8 mutation. Whole-cell current was recorded from VD5 by applying a series of voltage steps (-60 mV to +70 mV at 10-mV intervals) from a holding voltage of -60 mV. Shown are sample current traces and current-voltage relationships of wild type (*wt*) and *unc-8(tm2071)*. Sample size (*n*) = numbers of independently recorded cells. *n* = 10 in both groups. Data are presented as mean values ± SEM. Source data are provided as a Source data file.

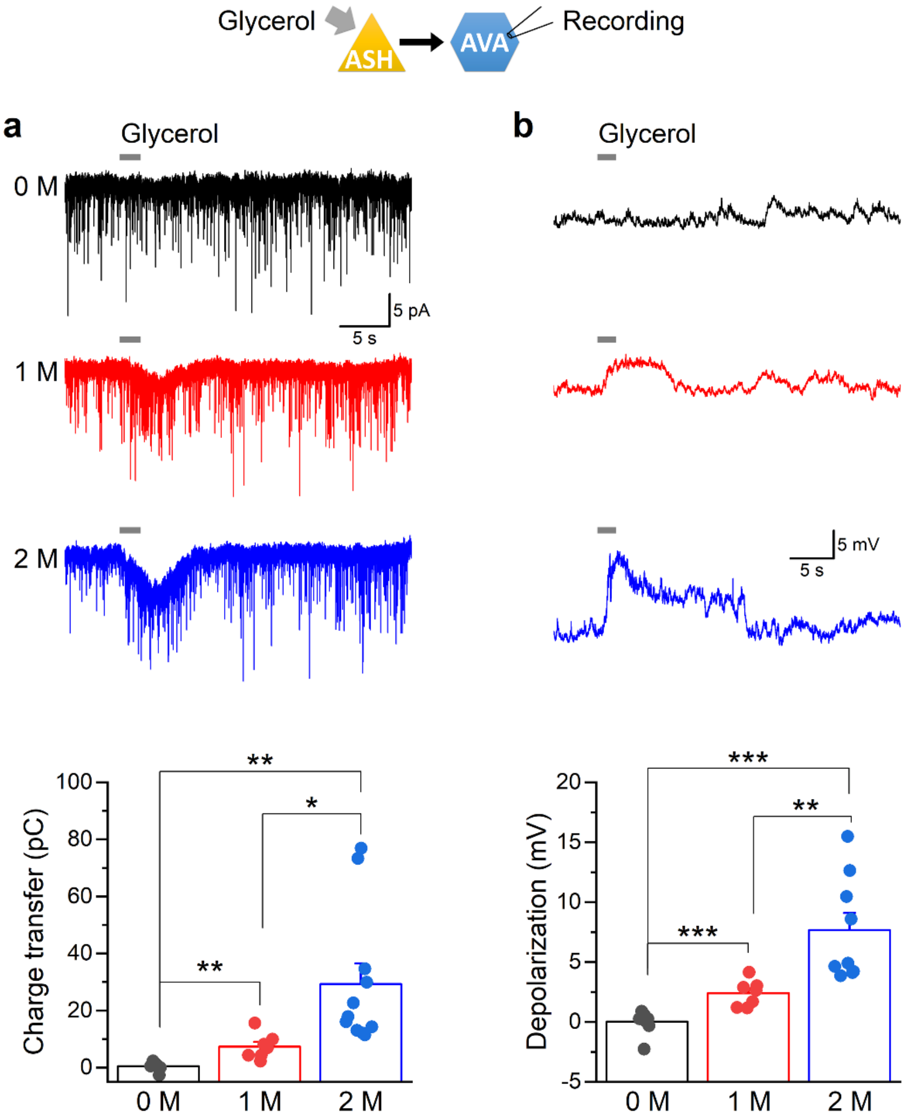

Supplementary Fig. 8. Application of hyperosmotic glycerol solutions to the vicinity of the nose activated AVA. a, Glycerol caused inward current in AVA (held at -60 mV) in a concentration dependent manner. Sample size (n) = numbers of independently recorded cells. n = 7, 7 and 11 at 0 M, 1 M and 2 M of glycerol, respectively. The p values are 0.002 (between 0 and 1 M), 0.006 (between 0 and 2 M), and 0.030 (between 1 and 2 M). **b**, Glycerol caused AVA membrane depolarization in a concentration dependent manner. n = 8, 7 and 9 at 0 M, 1 M and 2 M of glycerol, respectively. The p values are 0.001 (between 0 and 1 M), 0.000 (between 0 and 2 M), and 0.008 (between 1 and 2 M). Glyceral solutions were puffed through a glass pipette. The bath solution was used for puffing in the "0 M" experiment. The asterisks indicate significant differences between the indicated groups (* $p < 0.05$, ** $p < 0.01$, *** $p < 0.001$, one-way ANOVA with Tukey's post hoc test). Data are presented as mean values ± SEM. Source data are provided as a Source data file.

Supplementary Table 1. Primers used

Gene name	Sense	Antisense
P*unc-49*	AAGCTGCAGTGGGTCCGCCACATAAGAG	ATTACCGGTTTCTCGAGAATGGAGCTTCT
unc-9 RNAi	GAGGATCCAGGATGAGTATGCTATTGTATT	ATTCTCGAGCTGATTGCCAGCTGAGCAG
lgc-46 RNAi	ATGAACCCCTGTAAGTACAATTT	ATGTTACCAAACTGGAAAGTCAT
unc-17 RNAi	ATGGATGATTTTGGGGCTATGC	ATGGTGGGCTCGAGGAAGGC
unc-49 RNAi	TCTGGTACCATGGCTCGTCCATTCACACTTAT	TGTGCCGGCAGCTTTCAATTTCCAGTTTACAG
unc-8 RNAi	AATACCGGTATGTCACCTTTGCTGACGT	AATGCTAGCCTAGAATATCACCTCCTGGC
